# Evaluation of Effects of Various Irrigating Solutions on Chemical Structure of Root Canal Dentin Using FTIR, SEM, and EDS: An In Vitro Study

**DOI:** 10.3390/jfb13040197

**Published:** 2022-10-20

**Authors:** Indu Padmakumar, Dharam Hinduja, Abdul Mujeeb, Raghu Kachenahalli Narasimhaiah, Ashwini Kumar Saraswathi, Mubashir Baig Mirza, Ali Robaian, Syed Nahid Basheer, Mohmed Isaqali Karobari, Giuseppe Alessandro Scardina

**Affiliations:** 1 Department of Conservative Dentistry and Endodontics, SJM Dental College and Hospital, Chitradurga 577501, Karnataka, India; 2 Conservative Dental Science Department, College of Dentistry, Prince Sattam bin Abdulaziz University, AlKharj 11942, Saudi Arabia; 3 Department of Restorative Dental Sciences, College of Dentistry, Jazan University, Jazan 45142, Saudi Arabia; 4 Department of Conservative Dentistry & Endodontics, Saveetha Dental College & Hospitals, Saveetha Institute of Medical and Technical Sciences University, Chennai 600077, Tamil Nadu, India; 5 Department of Restorative Dentistry & Endodontics, Faculty of Dentistry, University of Puthisastra, Phnom Penh 12211, Cambodia; 6 Department of Surgical, Oncological and Stomatological Disciplines, University of Palermo, 90133 Palermo, Italy

**Keywords:** endodontics, irrigants, root dentin, sodium hypochlorite, silver citrate, ozonated olive oil, chemical properties

## Abstract

**Background:** Sequential chemical application for irrigating a root canal during chemomechanical debridement can affect the dentin microstructure. Understanding the effects of various irrigants on chemical properties of dentin can elucidate their effects on physical properties and thereby explain the higher incidence of structural failure in endodontically treated teeth. This in vitro research aimed to compare and evaluate the effects of three different irrigating solutions on the chemical structure of root canal dentin in extracted human teeth. **Methods:** Forty-eight extracted single-rooted mandibular premolar teeth were sectioned at the cemento–enamel junction by a diamond disc and were then randomly assigned to four groups of twelve samples each. The groups were irrigated using 5.25% NaOCl, ozonated olive oil, silver citrate, or distilled water. Dentin sections measuring 1.5 mm were obtained from the root portion and each section and were analyzed using Fourier transform infrared spectroscopy (FTIR), scanning electron microscopy (SEM), and electron-dispersive spectroscopy (EDS). FTIR and EDS values are reported as means ± standard deviations. Data were analyzed using an ANOVA and a post hoc Bonferroni test (*p* < 0.05). **Results:** A comparison of the FTIR and EDS values among the groups using ANOVA revealed statistically significant differences in the organic and inorganic peak values among the groups. An intergroup comparison between NaOCl with silver citrate and ozonated olive oil revealed significant reductions in the carbonate and phosphate peak values in the NaOCl group (*p* < 0.05). The EDS values tabulated for the carbon, oxygen, phosphorous, and calcium peak levels showed significant differences between the groups using an ANOVA. An SEM analysis was conducted under 1500× magnification, which revealed smear layer removal in the silver citrate group. **Conclusions**: The silver citrate solution and the ozonated olive oil caused less changes in the organic and mineral contents of dentin than sodium hypochlorite.

## 1. Introduction

Chemomechanical debridement plays an important role in the success of root canal treatments. This is performed by utilizing appropriate instruments along with effective irrigating solutions, followed by sealing with suitable materials [1]. A successful root canal treatment requires irrigation, as it fulfils several mechanical, chemical, and microbiological functions, including healing the periapical tissues. Irrigation thus plays a central role in endodontic treatment, though there is no single irrigant that efficiently satisfies all the required functions of an ideal irrigating solution [2]. The tissue dissolution capacity and the antimicrobial effect are two vital features of endodontic irrigants that enable them to play an integral part in chemomechanical root canal preparation. While enhancing the elimination of microbiota and facilitating the elimination of necrotic tissue and dentin debris from the root canal system, an ideal irrigant should also be nonirritant to the surrounding root and should not debilitate the tooth structure by causing excessive wear of minerals from the dentin [2,3].

Currently, a wide array of endodontic irrigants is available on the market, although sodium hypochlorite continues to be one of the most prominent and widely used endodontic irrigants. It is available in various concentrations ranging from 0.5 to 5.25% [4]. Sodium hypochlorite has been proven to be efficacious in dissolving organic compositions and pulp remnants, and it is the only irrigating solution that has the capability to dissolve vital and nonvital organic tissues [3]. However, it causes considerable destruction to the collagen of surface dentin within a relatively short period of time, which impairs the flexural and elastic strength of the dentin. Additionally, at the end of the chemomechanical preparation, irrigation with hypochlorite causes strong erosion of the surface dentin of the canal wall [5]. Hence, if it is not used judiciously, it could jeopardize the longevity of the root canal treatment. Due to increasing safety concerns, newer irrigants are being studied for the potential replacement of sodium hypochlorite.

In recent studies, a novel endodontic irrigating solution containing silver citrate was developed using electrolytically generated silver ions (0.003%) in citric acid (4.846%) and was tested as an innovative biomaterial for disinfecting and cleaning root canals. The patented aqueous disinfectant is a powerful antimicrobial agent that is produced by an electrochemical process using silver and citric acid that produce a stabilized silver ion complex, which develops a molecular complex, AgC_6_H_7_O_7_, by weakly bonding a silver ion to a citrate ion. This novel irrigant was reported to be nontoxic and biocompatible [6,7].

Ozone has numerous beneficial effects, including its antimicrobial activity, the oxidation of bacterial biomolecules and microbial toxins, the ability to remove the smear layer, and opening dentinal tubules to allow the deeper penetration of Ca and fluorine ions into them [8]. Ozonated oils are obtained by the means of chemical reactions that pass pure oxygen and ozone through the oils, and they are potent antibacterial irrigants [9]. Although previous studies have stated the antibacterial efficacy of ozonated olive oil and silver citrate in root canals, this is a pioneer study on its effect on the chemical structure of root dentin.

A variety of chemical irrigating solutions have been studied to determine their modes of action and effectiveness on both the organic and inorganic components of root canal dentin. These effects are directly related to the mechanical, chemical, and physical properties of the dentin structure [10]. Although very limited research has been conducted corelating the effects of various irrigants on root dentin, when considering the long-term success of a root canal treatment, maintaining the chemical and mechanical integrity of root dentin plays an integral role. Thus, it is important that novel irrigants be studied regarding their effects on the dentin microstructure so as to reduce the detrimental and erosive effects of conventional irrigants. This in vitro research aimed to compare and evaluate the effects of three different irrigating solutions on the chemical structure of root canal dentin in extracted human teeth. The null hypothesis states that these irrigating solutions do not alter the chemical structure of root dentin.

## 2. Materials and Methods

Ethical clearance for the study was obtained before the start of the study from the institutional ethics review committee with code (Ref. BMC&H/I EC/2021-22/32). This in-vitro study was conducted on 48 extracted single-rooted mandibular premolar teeth. They were extracted for the purpose of orthodontic treatment and were collected from the department of oral and maxillofacial surgery.

### 2.1. Sample Preparation

All collected samples were washed with distilled water and cleaned using ultrasonic scaling. Then, the specimens were stored in a chloramine-T solution. Soft tissues and debris were removed, and a diamond disc (Ray Foster, Huntington Beach, CA, USA) was used to decoronate the tooth at the level of the cemento–enamel junction.

Samples were then randomly assigned to four groups (Gp) with 12 samples each:

Group 1: 5.25% NaOCl;

Group 2: Ozonated olive oil (Ozonoid; Adc Inc. Dentozoneindia; Maharashtra, Mumbai, India);

Group 3: Silver citrate (BioAKT, New Tech Solutions s.r.l., Brescia, Italy);

Group 4: Distilled water.

A conventional access cavity preparation was created to access the root canal system. A size 10 K file was placed in each canal to determine its patency. According to the manufacturer’s instructions, the working length was set at 1 mm below the apex, and the canals were progressively enlarged up to size F2 Protaper Gold (Dentsply, Maillefer, Switzerland) while being irrigated with 5 mL of the appropriate irrigating solution in between each instrument, for a total of 20 mL of irrigating solution per tooth sample. The root portion was further sectioned into three parts using a diamond disc, and the middle third of the tooth was used for the experiment, as it presented with adequate root dentin thickness and uniform canal anatomy compared to the apical third. The middle third of the root portion was horizontally cut into slices of 1.5 mm thickness using a diamond disc.

### 2.2. Fourier Transform Infrared Spectroscopy (FTIR) Analysis

**Figure 1:**
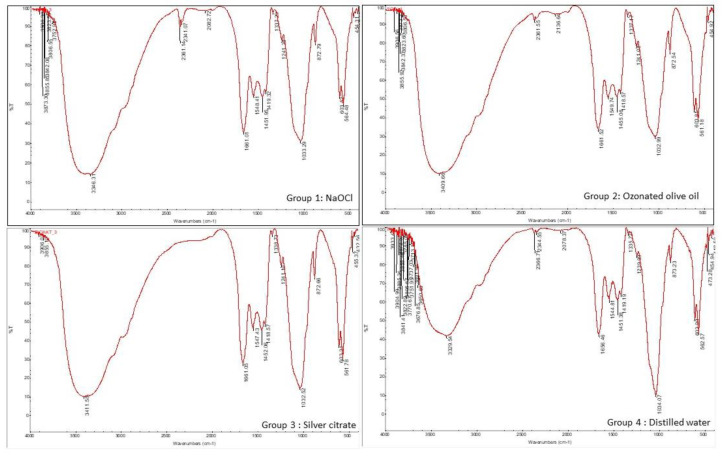
Collagen, phosphate, and carbonate peak levels, as determined by an FTIR spectrometer between 400 and 4000 cm at a 1 cm resolution using ten scans for groups 1, 2, 3, and 4.

Dentin discs were obtained from the middle third of the root and dentin slices of 1.5 mm thickness was obtained using a diamond disc. Samples were analyzed with an ATR-FTIR (iS50 Nicolet FTIR Spectrometer; ThermoFisher Scientific; Waltham, MA, USA) between 400 and 4000 cm at a 1 cm resolution over the course of ten scans following irrigation with the respective irrigants. Collagen, phosphate, and carbonate peak levels were determined (in cm^−1^) (Figure 1).

### 2.3. Scanning Electron Microscopy (SEM) and Electron-Dispersive Spectroscopy (EDS) Analysis

**Figure 2:**
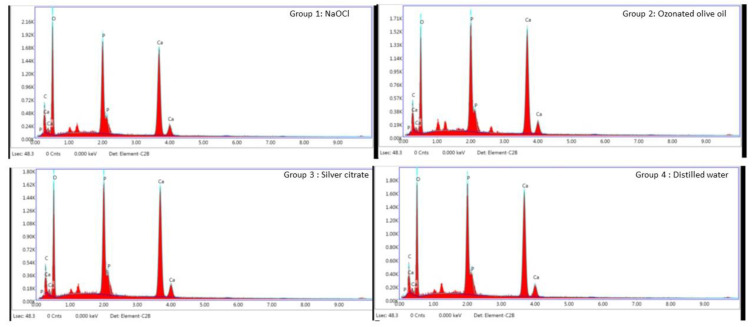
EDS analysis measured atomic percentages of carbon, oxygen, phosphorous, and calcium levels of dentin samples obtained from group 1, 2, 3, and 4 using iridium software at 20 KeV.

The same samples were dried at 37 °C for 48 h, and sample segments were fixed in stubs with the dentin walls upwards. The samples were coated with two thin layers of evaporated carbon in high vacuum by a desk carbon coater and viewed at a magnification of 1500× with a field-emission SEM (JEOL IT-300, JEOL. Ltd., Tokyo, Japan). To measure the atomic percentages of the carbon, oxygen, phosphorus, and calcium levels in the dentin samples, an EDS analysis was carried out in conjunction with SEM using iridium software at an accelerating voltage of 20 KeV (Figure 2).

### 2.4. Statistical Analysis

SPSS (Statistical Package For Social Sciences) version 20 (IBM SPASS statistics (IBM Corp.) released 2011) was used to perform the statistical analysis. Inferential statistics such as ANOVA were applied to check the statistical differences in chemical structural changes among the groups, with post hoc Bonferroni for intergroup comparisons.

## 3. Results

**Figure 3:**
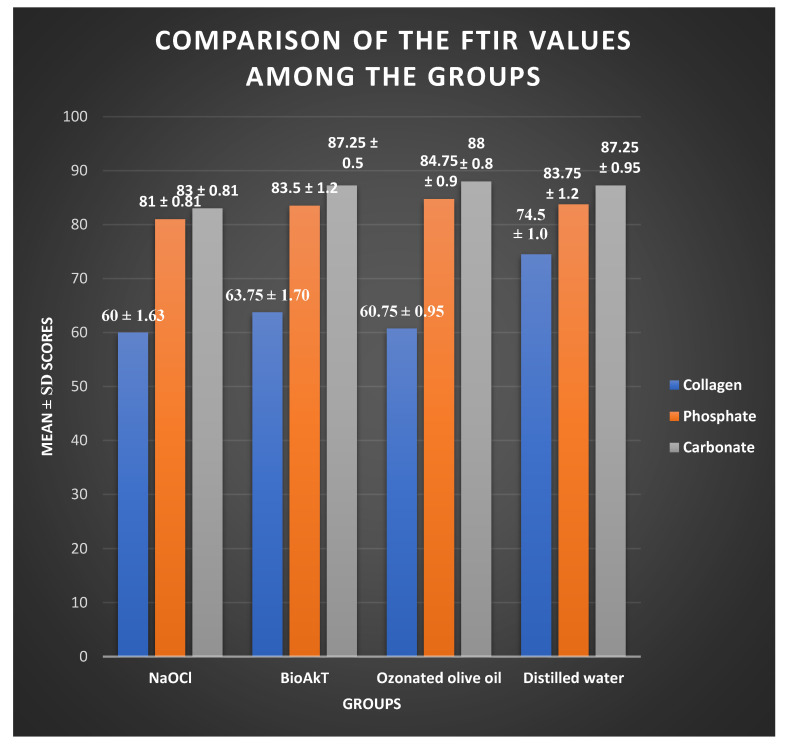
The collagen, phosphate, and carbonate peak values obtained by FTIR analysis for group 1: NaOCl (sodium hypochlorite), group 2: silver citrate, group 3: ozonated olive oil, and group 4: distilled water.

The comparison of the FTIR values among the groups using ANOVA revealed statistically significant differences in the collagen carbonate and phosphate peak values among the groups (Figure 1). A decrease in the collagen level was observed in the NaOCl group compared to the experimental and negative control groups, although the results were not statistically significant (*p* > 0.05). The intergroup comparisons between NaOCl and silver citrate as well as ozonated olive oil revealed significant reductions in the carbonate and phosphate peak values in the NaOCl group (*p* < 0.05) (Figure 3).

**Figure 4:**
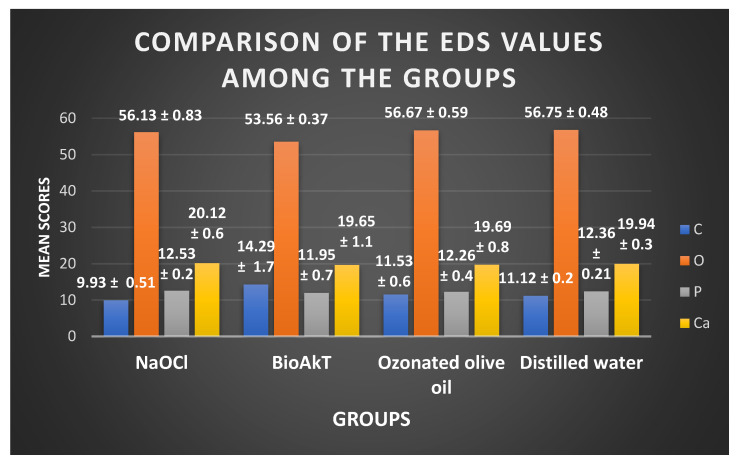
The inorganic mineral content of dentin, as obtained from EDS analysis, after irrigation with the respective irrigants. C—carbon, O—oxygen, P—phosphorous, C—calcium.

The EDS values tabulated for the carbon, oxygen, phosphorous, and calcium peak levels showed significant differences between the groups using an ANOVA (Figure 4). The mean value for the carbon peak levels in NaOCl was significantly different compared to the silver citrate and ozonated olive oil. Silver citrate showed reduced mean values for Ca and P compared to the other groups, although they were not statistically significant (*p* > 0.05) (Figure 4).

**Figure 5:**
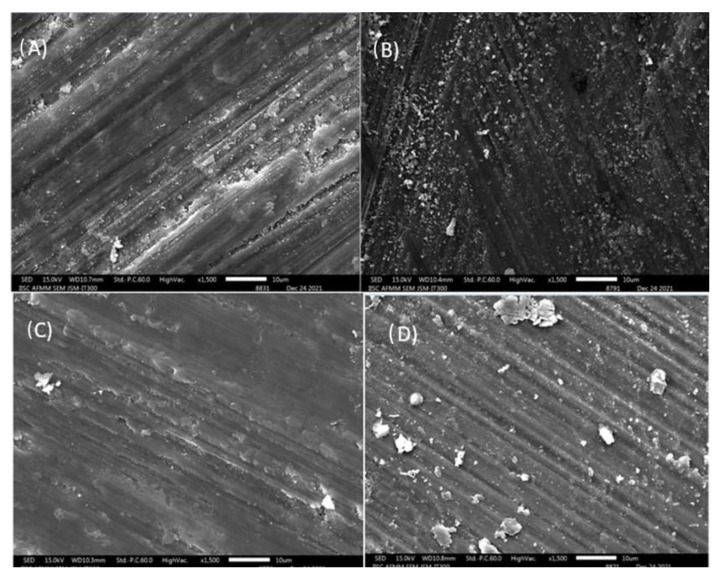
SEM image showing dentin cross sections under 1500× magnification after irrigation with (**A**) sodium hypochlorite, (**B**) ozonated olive oil, (**C**) silver citrate, and (**D**) distilled water.

The SEM analysis was performed under 1500× magnification and revealed smear layer removal in the silver citrate group. The partial removal of the smear layer was observed in the sodium hypochlorite group, while ozonated olive oil and distilled water did not exhibit smear layer removal (Figure 5).

## 4. Discussion

Our ability to strike a balance between the “biological” goals and the “mechanical” objectives of the therapy continues to be the clinical challenge of root canal therapy. The ideal irrigation protocol is one that eliminates biofilm for maximum antibacterial effectiveness while having no negative effects on the mechanical integrity of the tooth. The damaged root canal filling–dentin interface during treatment further complicates this requirement. It is important to take into account how endodontic irrigants directly alter the chemomechanical characteristics of the root canal dentin, which has an impact on the effectiveness and durability of all materials used for root canal obturations and restorations.

In the current study, various novel endodontic irrigating solutions were tested individually to determine their effects on dentin through various methods of chemical analysis, including elemental (EDS) and chemical (FT-IR) determinations of the dentin constitution and a surface microstructural analysis by SEM. The null hypothesis was rejected, as various endodontic irrigants used in this study altered the chemical structure of root dentin.

NaOCl significantly reduced the organic components of the dentin such as carbonate and phosphate compared to novel irrigating solutions such as silver citrate and ozonated olive oil. This is in accordance with studies conducted by Sakae et al., who stated that NaOCl is capable of removing magnesium and carbonate ions from dentin [11]. All the irrigating solutions were shown to affect the collagen content of the root dentin, although NaOCl showed the highest mean difference compared to distilled water. NaOCl at a concentration of 1.5% was shown to reduce the collagen content and caused a subsequent reduction in flexural strength, which is in agreement with the observations of this study [12]. At higher concentrations ranging from 5 to 9%, as used in this study, it can cause alterations in the carbon and nitrogen contents of dentin, reducing the dentin microhardness, as reported by Marending et al. The organic-tissue-dissolving properties of sodium hypochlorite as an irrigant on the collagen component of dentin has already been established by various studies, and it has been shown to affect the microhardness of dentin [13,14,15].

NaOCl spreads on the intrafibrillar water volume of apatite-encapsulated collagen matrix owing to its low molecular weight. Collagen from the “superficial subsurface” of mineralized dentin undergoes oxidative chemical destruction when it comes into contact with sodium hypochlorite. According to Huang et al., dentin specimens can lose their toughness and flexural strength with just a 1 μm depth of collagen degradation on the dentin surface [16].

The silver citrate solution used in the study comprised 4.8% citric acid. Previous studies have stated that citric acid at concentrations between 25% and 50% is an effective endodontic irrigant. However, recent studies have demonstrated the efficacy of citric acid solutions with lower concentrations (<10%) to be operational. In comparison to the other groups, the silver citrate group displayed a slightly larger quantity of calcium dissolution, which is consistent with citric acid’s capacity to decalcify hard dental tissues by the chelation of Ca^2+^ ions in a mildly acidic environment [17].

Aldehydes, ketones, and hydrogen peroxide can be generated as a result of the hydrolysis of ozonized oil. As an oxidant, hydrogen peroxide degrades vital biological components such as lipids, proteins, and nucleic acids [18]. Hydrogen peroxide also has an effect on the inorganic components of dentin by acidic demineralization [19]. Silver citrate (BioAKt) exhibited effective smear layer removal compared to the other groups in the study. Several studies have stated the efficiency of citric acid in smear layer removal, which was superior to EDTA at similar concentrations [20]. It has been suggested that irrigant solutions with the ability to remove the smear layer may reduce dentin microhardness; however, when these solutions are used for a brief period of time inside the root canal, this reduction does not appear to have a negative impact on the fracture resistance of teeth that have undergone endodontic treatment [21]. All irrigating solutions used in this study were only briefly in contact with the dentin, which may account for the comparable mineral compositions of the dentin seen in the experimental groups (Figure 4). Furthermore, the exposure time of irrigants to the dentinal wall has been standardized, as it could have an impact on the elemental composition of carbon, calcium, phosphorous, and oxygen [10].

The mechanical, physical, and chemical features of root dentin must be conserved against any harmful impacts of chemical substances [22]. However, it should be emphasized that this study has some limitations that should be addressed. One of the main limitations of this study is that it was conducted in vitro, thus failing to simulate the conditions of the oral cavity. Future research is needed to evaluate the changes in physical properties upon the usage of these irrigants and to correlate them with the results of this study. In addition, further research with the criteria used in this study are encouraged to emphasize the relevance of the findings.

## 5. Conclusions

It was apparent that all studied irrigation sequences potentially result in some alteration in the inorganic and organic contents of the dentin. Furthermore, it can also be concluded from the results that the silver citrate solution and the ozonated olive oil cause fewer changes in the organic and mineral contents of dentin than sodium hypochlorite.

## Data Availability

The data will be made available by the corresponding author on reasonable request.
